# The incidence and geographical spread of SARS-CoV-2 in Rio de Janeiro, Brazil based on RT-PCR test results

**DOI:** 10.1590/0037-8682-0779-2020

**Published:** 2021-02-10

**Authors:** Guilherme Loureiro Werneck, Luís Cristóvão Porto, Alexandre Sena, Orlando da Costa Ferreira, Andrea Cony Cavalcanti, Ângela Maria Guimarães Santos, Danielle Angst Secco, Marcio Silva, Diana Mariani, Alexandre Chieppe, Amilcar Tanuri, RTPCR-RJ Working Group

**Affiliations:** 1 Universidade do Estado do Rio de Janeiro, Departamento de Epidemiologia, Rio de Janeiro, RJ, Brasil.; 2 Universidade Federal do Rio de Janeiro, Instituto de Estudos em Saúde Coletiva, Rio de Janeiro, RJ, Brasil.; 3 Universidade do Estado do Rio de Janeiro, Instituto de Biologia Roberto Alcântara Gomes, Rio de Janeiro, RJ, Brasil.; 4 Universidade do Estado do Rio de Janeiro, Instituto de Matemática e Estatística, Rio de Janeiro, RJ, Brasil.; 5 Universidade Federal do Rio de Janeiro, Instituto de Biologia, Rio de Janeiro, RJ, Brasil.; 6 Secretaria de Estado de Saúde do Rio de Janeiro, Laboratório Central de Saúde Pública Noel Nutels, Rio de Janeiro, RJ, Brasil.; 7 Secretaria de Estado de Saúde do Rio de Janeiro, Subsecretaria de Vigilância em Saúde, Rio de Janeiro, RJ, Brasil.

**Keywords:** SARS-CoV-2, COVID-19, Epidemiology, Surveillance, Brazil

## Abstract

**INTRODUCTION:**

Rio de Janeiro has hardly experienced coronavirus disease.

**METHODS:**

Here, 87,442 reverse transcription polymerase chain reaction (RT-PCR) test results for severe acute respiratory syndrome coronavirus 2 (SARS-CoV-2) were reported among Rio de Janeiro residents (March to September 2020).

**RESULTS:**

Overall, RT-PCR positivity of 44.6% decreased over time towards 20%. Positivity was greater among males (OR=1.22; 95%CI:1.19-1.26); Black (OR=1.10; 95%CI:1.02-1.19), Brown (OR=1.16; 95%CI:1.10-1.22), and indigenous people (OR=2.11; 95%CI:0.88-5.03) compared to Whites and increased with age; with epidemic spread from the capital to inland regions.

**CONCLUSIONS:**

SARS-CoV-2 keeps spreading in Rio de Janeiro, and reopening of activities may fuel the epidemic.

In Brazil, epidemiological data regarding the new coronavirus disease (COVID-19) pandemic are gathered from different sources and collated by the Surveillance Department of the Brazilian Ministry of Health^1^. By October 26, 2020, Brazil ranked third in the number of COVID-19 cases (5,409,854) and second in the number of deaths (157,397) worldwide^2^. 

The state of Rio de Janeiro has hardly experienced the COVID-19 pandemic, and the community transmission of the severe acute respiratory syndrome coronavirus 2 (SARS-CoV-2) has been declared since March 13, 2020^3^. By October 26, 2020, the state of Rio de Janeiro had 302,746 confirmed cases of COVID-19, ranking second in mortality rate (117 deaths per 100,000 population) and first in case-fatality rate (6.7%) in Brazil^1^ ^,^ ^2^.

The epidemiological situation in the state of Rio de Janeiro is of major concern essentially because it encompasses the city of Rio de Janeiro. Rio de Janeiro is the second largest city in Brazil, with more than 6.7 million people, 22% of whom live in densely inhabited slums under poor sanitary and household conditions^4^. The city of Rio de Janeiro was one of the first cities in Brazil to confirm COVID-19 cases. Today, for the city, the mortality rate of COVID-19 (177 deaths per 100,000 population), is higher than that of any other country, globally, and is the highest among Brazilian cities with more than 100,000 population^1^. The case-fatality rate is 10.2%, 3.5-fold higher than the country figure, suggesting that underreporting of cases might be substantial^1^. Therefore, understanding the spread of SARS-CoV-2 infection in the state of Rio de Janeiro and its capital might be useful to plan the next steps in the pandemic response not only locally but also in the country. This is because, the city of Rio de Janeiro, together with São Paulo City, are hubs for the spread of the disease to other regions in Brazil^3^.

The Health State Secretary of Rio de Janeiro routinely offers the molecular diagnosis for airborne viral infections through its Central Laboratory (LACEN). Since the beginning of 2020, the demand for testing has increased substantially, from 163 tests in February 2020 to 4,524 tests in March 2020. Furthermore, new collection sites for real-time reverse transcription polymerase chain reaction (RT-PCR) for SARS-CoV-2 were implemented. By the beginning of September 2020, more than 100,000 tests were performed and registered in the Laboratory Information System (GAL) of the Brazilian Ministry of Health^1^. 

Using the GAL database, we report 87,442 RT-PCR test results for SARS-CoV-2 among the state of Rio de Janeiro residents from March 10 to September 5, 2020, corresponding to the number of the initial tests of subjects referred for testing. Excluding 1,152 (1.3%) tests with an undetermined result, the total number of RT-PCR tests with valid results was 86,290, with a positivity rate of 44.6% (95% confidence interval [95%CI]: 44.2-44.9). 

Figure 1 shows the number of positive and negative tests and the percentage of positivity by epidemiological week. The percentage of positivity decreased slowly, reaching 20% by epidemiological week 36 (August 30 to September 5, 2020; p-value for trend <0.001). The decrease in RT-PCR positivity over time may represent both a gradual decline in the number of cases and deaths in the state of Rio de Janeiro since late May 2020, and the increased availability of tests for mild symptomatic and asymptomatic persons^5^ ^,^ ^6^. 

**FIGURE 1:**
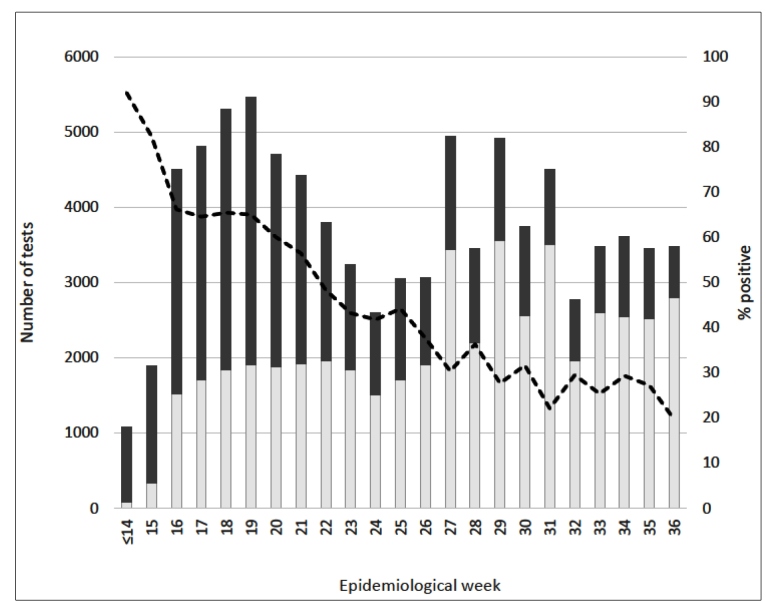
Number of positive and negative test results and the percent of positivity by epidemiological week, the state of Rio de Janeiro, Brazil, 2020.

In Table 1, we present the prevalence of RT-PCR positivity rates and associated factors including sex, age group, race/skin color, and region of residence. Association between variables and RT-PCR positivity was expressed as odds ratio (OR) and respective 95%CI obtained by logistic regression models (Stata version 15.1, StataCorp LLC, College Station, TX, USA). Confidence intervals and p-values were adjusted for multiple testing using the Sidak procedure (Stata version 15.1). The state of Rio de Janeiro is divided into 10 regions (9 health regions plus the city of Rio de Janeiro). By September 5, 2020, the prevalence of COVID-19 RT-PCR positivity ranged from 26.4% (95%CI 24.7-28.1) in the North region of the state to 52.6% (95%CI: 51.8-53.6) in the Metropolitan I region (Table 1). Positivity was significantly greater among males (47.4%) than in females (42.2%, OR = 1.22; 95%CI: 1.19-1.26) and increased monotonically with age (p-value for trend <0.001) (Table 1). Data on race/skin color were available only for 57.6% of the patients, and RT-PCR positivity was higher among Blacks (42.7%, OR = 1.10; 95%CI: 1.02-1.19), Brown (44.0%, OR = 1.16; 95%CI: 1.10-1.22), and indigenous people (58.8%, OR = 2.11; 95%CI: 0.88-5.03) than among Whites (40.3%) (Table 1). 

**TABLE 1:** Prevalence of positivity in RT-PCR and associated factors, state of Rio de Janeiro, Brazil, 2020.

Variables	N	Prevalence (%)	Odds ratio	95%CI	p-value
Sex					
Female	48,616	42.2	-		
Male	38,586	47.4	1.22	1.19-1.26	<0.001
Age group (years)					
<10	2,015	15.0	-		
10-19	2,228	22.2	1.61	1.38-1.89	
20-29	11,055	34.5	2.98	2.62-3.39	
30-39	17,907	42.4	4.16	3.67-4.72	<0.001*
40-49	17,165	45.8	4.78	4.21-5.42	
50-59	14,140	47.7	5.15	4.54-5.85	
60-69	10,561	51.7	6.07	5.34-6.89	
70+	12,168	53.8	6.59	5.80-7.49	
Race / skin color					
White	23,811	40.3	-		
Black	5,832	42.7	1.10	1.02-1.19	0.002
Brown	14,653	44.0	1.16	1.10-1.22	<0.001
Asian	5,378	40.2	0.92	0.94-1.07	1.000
Indigenous	34	58.8	2.11	0.88-5.03	0.125
Region of residence					
North (*Norte*)	2,666	26.4	-		
Northwest (*Noroeste*)	4,009	29.6	1.17	1.00-1.36	0.041
South Center (*Centro-Sul*)	4,030	31.7	1.29	1.11-1.51	<0.001
Middle Paraíba (*Médio Paraíba*)	11,368	37.5	1.66	1.46-1.90	<0.001
Mountains (*Serrana)*	6,697	37.9	1.70	1.47-1.95	<0.001
Coastal Lowland (*Baixada Litorânea*)	7,526	40.6	1.90	1.65-2.18	<0.001
Ilha Grande Bay (*Baía da Ilha Grande*)	1,645	46.5	2.42	2.01-2.91	<0.001
Rio de Janeiro city	28,239	50.5	2.84	2.50-3.22	<0.001
Metropolitan II (*Metropolitana II*)	7,303	50.5	2.84	2.47-3.25	<0.001
Metropolitan I (*Metropolitana I*)	12,791	52.6	3.09	2.71-3.53	<0.001

*Chi-square test for trend.

Figure 2 shows the positivity rate per 100,000 population for each municipality, and the proportion of positive and negative tests in each state region for seven calendar periods. The positivity rates in each municipality were spatially smoothed using the local empirical Bayesian estimation procedure with a contiguity queen matrix (TerraView 3.3.1, Instituto Nacional de Pesquisas Espaciais, São José dos Campos, SP). At the beginning of the epidemic (March 2 to March 29, 2020), the positivity rate per 100,000 population did not substantially vary between the regions, and most results were positive, suggesting that testing was concentrated among the most symptomatic patients (Figure 2, panel B). However, the rates of positivity started to increase in subsequent periods in all regions. However, particularly in the city of Rio de Janeiro and the neighboring metropolitan areas, the percentage positivity was between 60% and 75%, while remaining less than 50% in most of the other regions (Figure 2, panels C-D). Between April 27 and May 24, 2020 (Figure 2, panel E), the positivity rates decreased in Rio de Janeiro City and the metropolitan areas, increasing mainly in their adjacent regions (Centro-Sul, Baixada Litorânea, Baia de Ilha Grande, and Médio Paraíba). The percent positivity was about or less than 50% in all other regions except for the Baia de Ilha Grande region (Figure 2, panel E). From June 22, 2020 onwards, the positivity rates substantially decreased in Rio de Janeiro City and the metropolitan areas, while the infections spread to the more distant Serrana and Noroeste regions of the state (Figure 2, panels F-H). It is worth noting that the North region maintained relatively low rates of positivity over the entire period. A dynamic visualization of the RT-PCR results and positivity rates per 100,000 inhabitants in each region is available at http://mnps.dev.br/.

**FIGURE 2:**
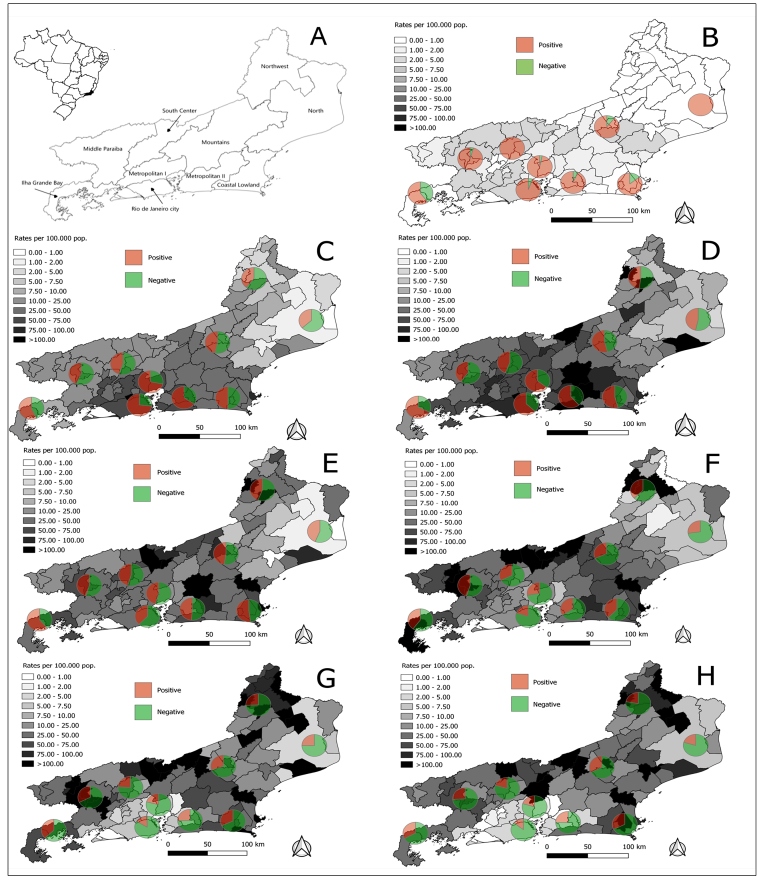
A) The state of Rio de Janeiro in the context of Brazil; B) to H) pie charts represent the number of positive (red) and negative (green) test results in each of the state regions in seven calendar periods. The positivity rates per 100,000 persons in each municipality were spatially smoothed using the local empirical Bayes estimation. B) Data from March 2 to March 29, 2020. C) Data from March 30 to April 26, 2020. D) Data from April 27 to May 24, 2020. E) Data from May 25 to June 21, 2020. F) Data from June 22 to July 19, 2020. G) Data from July 20 to August 16, 2020. H) Data from August 17 to September 13, 2020.

The greater RT-PCR positivity rate among the males may be related to mobility and lifestyle. However, biological differences cannot be disregarded as potential explanations^7^ ^,^ ^8^. This result is of great concern since men have about twice the risk of death from COVID-19, although women are more likely to bear the brunt of the social and economic consequences of the pandemic^9^. The increasing RT-PCR positivity rate with age may be related to both the increased mobility of people in the workforce and the higher susceptibility of the elderly in developing symptomatic infection^10^ ^,^ ^11^. Interpretations of the racial differences in RT-PCR positivity must be considered with caution because of the high levels of missing values in this variable. However, racial differences in positivity may reflect social inequalities mediated by structural racism in a country that never eliminated its colonial tradition^12^. Specifically, the greater test positivity rates among the Blacks, Brown, and indigenous people might have originated from the racial inequalities in the access to RT-PCR testing. The lower positivity rate among Whites might occur because they are more likely to have access to testing when they have only mild symptoms, a condition associated with a lower RT-PCR sensitivity. In addition, mild symptoms might not even be caused by SARS-CoV-2 infection. Conversely, the Blacks, Brown, and indigenous people may be tested only when presenting with more severe symptoms, in which case, they are more likely to have a positive RT-PCR result. 

The geographical variations in RT-PCR positivity indicates the spread of the epidemic from the city of Rio de Janeiro to inland regions. The decrease in the currently observed number of cases and deaths in the state of Rio de Janeiro is not uniform across the regions, with the city of Rio de Janeiro and the Metropolitan I region showing stabilization since August, followed by a recent increase^5^ ^,^ ^13^. Social, demographic, and cultural factors vary geographically, and differences in the levels of enforcement of social distancing recommendations may determine, at least partially, the heterogeneous trends of the COVID-19 incidence in the state. Such situations require epidemiological interpretations that consider regional mobility patterns and adherence levels to preventative measures to better define local control strategies^14^.

The data presented herein have some limitations that should not be overlooked. First, the data did not include RT-PCR test results from private laboratories and minor public institutions. However, this missing data represent a negligible number of the total RT-PCR tests performed in the state of Rio de Janeiro in this period. Second, since nasopharynx swabs for children were not always available, some testing sites offered RT-PCR only for adults, leading to a skewed age distribution of the sample towards older ages. However, the proportion of positive tests in adults and children should not have been substantially affected by the underrepresentation of children in the sample. Finally, it is not easy to ascertain the actual RT-PCR testing coverage, as some localities may not have been reached.

This is the first and largest report of RT-PCR results by age, sex, time, and region from the state of Rio de Janeiro during the COVID-19 pandemic. Such analysis could inform surveillance strategies and testing efforts, allowing public health authorities to tailor prevention strategies oriented to those most at risk^15^. Indeed, future vaccination campaigns against COVID-19 are likely to suffer from insufficient vaccines for the entire population of Rio de Janeiro. Therefore, identifying demographic characteristics associated with a higher risk of infection and delimiting geographic areas with high SARS-CoV-2 transmission could be used to help define priority groups for vaccination.

The state of Rio de Janeiro and the Health Ministry public authorities face the challenge of interpreting the trends in reported cases and deaths, and data from RT-PCR and serologic studies to decide the best strategy for dealing with the epidemic. Similarly, most municipalities in the state of Rio de Janeiro started a rapid process of relaxing social distance measures and reopening commercial activities. There is a high degree of underreporting, delays between testing and confirming of infections, as well as a suboptimal surveillance structure for identifying symptomatic cases and tracing contacts. Therefore, one should consider that the recent decrease in the number of cases and deaths may soon be followed by an increase in the transmission. Regardless, the RT-PCR positivity rate of around 20% was too high. This shows that SARS-CoV-2 keeps spreading in the state of Rio de Janeiro at an alarming rate, and the reopening of all activities may fuel a new burst of infections.
