# Erratum

**DOI:** 10.1590/0037-8682-0779b-2020

**Published:** 2021-04-12

**Revista da Sociedade Brasileira de Medicina Tropical/*Journal of the Brazilian Society of Tropical Medicine***

**Title:** The incidence and geographical spread of SARS-CoV-2 in Rio de Janeiro, Brazil based on RT-PCR test results

**Vol. 54(e07792020): 2021 - Page: 01, - doi:** 10.1590/0037-8682-0779-2020

**Abstract/Introduction:**

Rio de Janeiro has hardly experienced coronavirus disease.

**Should read:**

 Rio de Janeiro has been strongly hit by coronavirus disease.

